# Quick Oil Red O, Paint3D and Biopython as an economical tool to measure total lipid levels in *Caenorhabditis elegans*

**DOI:** 10.17912/micropub.biology.001320

**Published:** 2025-01-14

**Authors:** Wenqing Wang, Elizabeth R Wong, Michael L Cato, Patrick C Daly, Chandler G Doody, Townshend Gillespie, Caroline N Ksor, Arooj Zainab, Zunaira Zainab, Matt Crook

**Affiliations:** 1 Stanford University, Stanford, California, United States; 2 Whitman College, Walla Walla, Washington, United States; 3 Wofford College, Spartanburg, South Carolina, United States; 4 Emory and Henry College, Emory, Virginia, United States; 5 Furman University, Greenville, South Carolina, United States

## Abstract

Quick Oil Red O staining is a well established method to assay total lipid levels in *Caenorhabditis elegans* , but the software to clean up and analyse the images is either laborious, expensive or both. We have developed a process that uses an existing protocol to stain the animals, followed by Magic Select in Paint3D to remove background and then a custom script in Biopython to quantify average pixel intensity *per* animal. The software is free, accessible and relatively easy to use for undergraduate researchers.

**Figure 1. qORO lipid staining and image analysis workflow:**
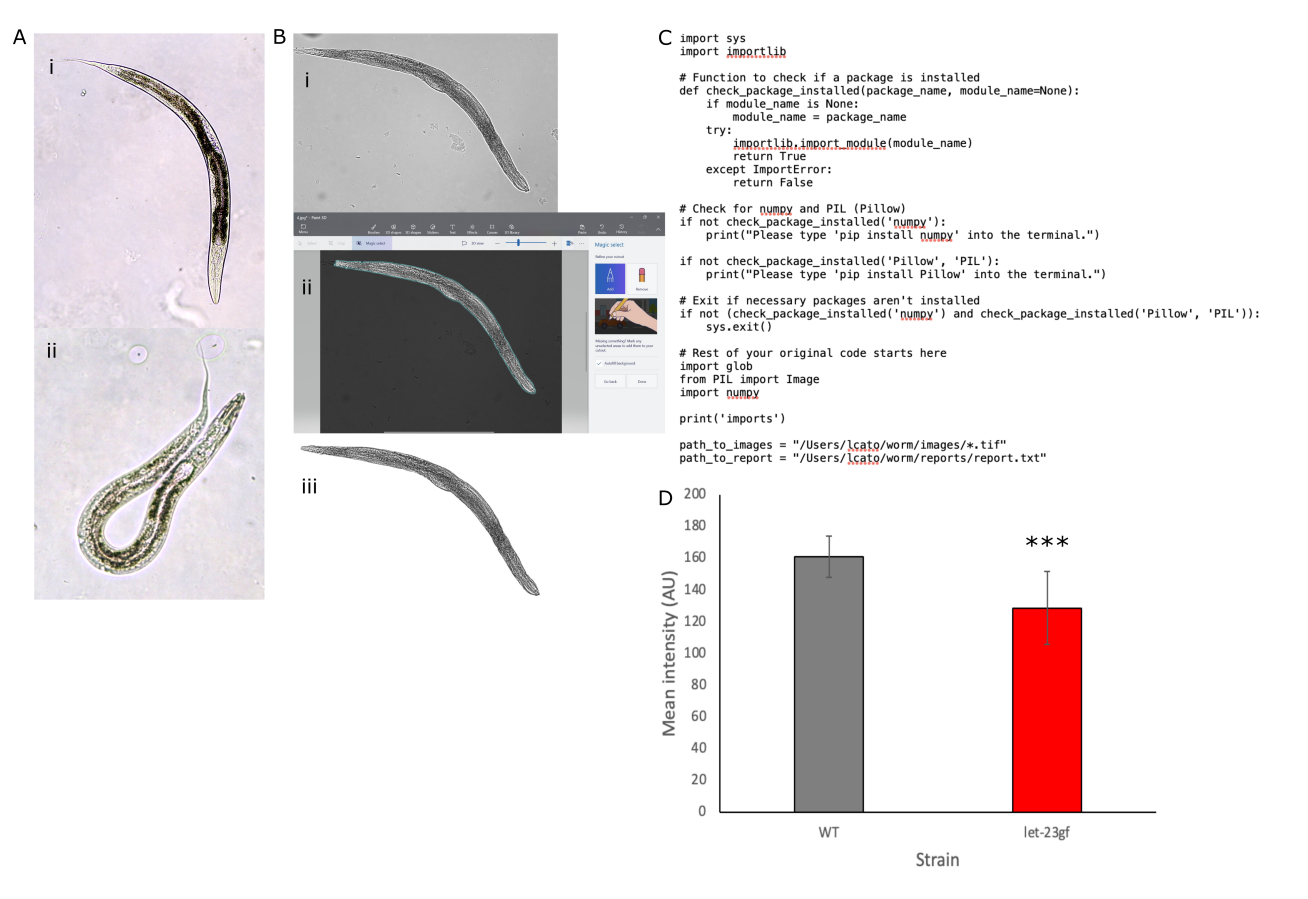
A) Representative colour images of (i) wild-type N2 and (ii) *let-23(sa62)gf* L4s. B) Background removal: i) original black and white brightfield micrograph, ii) selection of animal using Magic Select in Paint 3D and iii) final micrograph without background. C) part of the worm.py script we used to analyse our processed images. D) Mean pixel intensity (arbitrary units), representing total imaged lipid levels, was significantly lower in *let-23(sa62)gf* animals when compared with wild-type N2 animals using Student's T-test. Error bars represent +/- 1 S.D., asterixes represent p=0.0003.

## Description

Measuring whole animal lipid levels is a useful tool to study the effect of internal and external factors on metabolism. *Caenorhabditis elegans* is an excellent organism for whole animal lipid staining as it is small, transparent and easy to stain for total lipids. Nile Red and quick Oil Red O (qORO) are the main methods for lipid staining in *C. elegans* (Stuhr et al 22). We used qORO (Wahlby et al 14) as it is simple, robust and easy for undergraduate researchers to learn. In addition to staining and imaging, we needed a method to process and analyse the images that was free, reliable and accessible. We found that the Magic Select function in Windows Paint3D enabled a fast and robust removal of image background that did not require manually outlining the animal, one of the drawbacks of using ImageJ or Photoshop. A custom Biopython script converted those processed images into mean pixel brightness values in batches of thirty or more images. Separating the different steps of our approach, especially the image processing and image analysis, also allowed different undergraduates to work on the same project at times that suited their individual schedules. We were then able to use these mean pixel brightness data to compare *let-23(sa62)gf* mutant animals to wild-type Bristol N2 animals.

Our protocol consists of five steps: worm culture, qORO staining, image capture, background removal and image analysis. Worm culture and low throughput quick Oil Red O staining were carried out as previously described (Wahlby et al. 2014). Fig. 1A shows representative true colour images of wild-type Bristol N2 and *let-23(sa62)gf* animals.

Image capture

Late L4 and young adult hermaphrodites were picked from the mixed stage stained animals based on size and absence of eggs, as eggs stain very strongly with qORO. The selected animals were mounted on 5% agarose pads in M9 and photographed in black and white under brightfield using an OLYMPUS BX53 microscope at 20x magnification ( Fig. 1B i). Earlier larval stages may need to be photographed at 40x magnification. The condenser and light brightness were set to the same level for each slide. Images were captured using the CellSens software and a 1s exposure. Only images of animals with clear space around them and no underlying bubbles or artifacts were captured. Photomicrographs were saved as both .tif and .jpg files.

Background removal (Windows only)

We used the Magic Select function of Paint 3D to remove the background from our images ( Fig. 1B i). Unfortunately, Paint 3D is Windows only, although Photoshop can be used to achieve a similar effect on a Mac. After opening an image click the *Magic Select* icon in the menu bar next to a crop button. Once selected, crop the image around what you want to keep. Click *Next* and the program should automatically select what you want to keep ( Fig. 1B ii). However, if background artifacts are included with your animal or parts of it were not selected, you can use the *Add or Remove* to adjust the selection until it accurately outlines the entirety of the worm. Click the *Done* button and your image will be removed off the background allowing you to move it to the side of the canvas. The background can then be deleted by clicking and dragging over the entire canvas area and press delete. Move your image back onto the canvas and adjust so that the entire image is in the frame and save the image by selecting *Menu > Save as > Image* ( Fig. 1B iii).

Image analysis

Detailed instructions and the Biopython script are available at https://github.com/thewanderingprofessor2/Quick-oil-red-O-software-analysis-package . Image analysis consists of installing Biopython and modifying the script ( Fig. 1C ), moving the processed images to Biopython Images folder, running the worm.py script in Biopython and then copying the data from *Reports* into your spreadsheet program of choice. Installation of Biopython on Mac and Windows computers is quite different and instructions for both are provided. Once the Biopython is installed and the script *worm.py* is downloaded, create a folder (we call ours *Worm* ) on your computer and inside that folder create two folders, one titled *Images* and the other *Reports.* Open *worm.py* in a text editor and edit the file paths so they match the correct locations on your computer (username that must be changed is bolded and underlined below). For example:

path_to_images = "/Users/ **lcato** /Worm/Images/*.jpg"

path_to_report = "/Users/ **lcato** /Worm/Reports/report.txt"

If you prefer to use TIF files, change the file extension in “path_to_images” to “/*.tif”. Note that some image editing programs save .jpg files as .jpeg, so the file extension in *worm.py* needs to exactly match that of your files.

Then change “pic” and “bg_mean” to match the resolution of your images (the values that need to be changed are bolded and underlined below). For example:

pic = Image.new('I', ( **2048, 1536** )

and

bg_mean = bg_total / (( **2048.0** * **1536.0** ) - area)

Save your copy of *worm.py* . The images you want to have analyzed must be in the *Images* folder and are in the format specified in the code above. Depending on your computer, you can run the script through Terminal or by clicking on the *worm.py* icon itself. Instructions on how to do so on both Windows and Macintosh computers are described in detail in the help files on the Github page linked above. It will take each image, invert each pixel and calculate total pixel brightness, worm area and mean pixel brightness. No modifications to the script should be needed for different size animals or different levels of staining. After the script has finished analysing all the images in your *Images* folder a *report.txt* file will appear in your *Reports* folder, which can be opened in a text editor or Excel. The third column contains the mean pixel brightness, where a higher value represents a higher amount of qORO staining. The mean pixel brightness values can then be analysed in Excel, Google Sheets or similar program.

We used our protocol to compare images of twelve N2 L4s with twelve *let-23(sa62)gf* L4s. We found that lipid levels, represented by mean pixel intensity in arbitrary units, were significantly lower in *let-23(sa62)gf* animals ( Fig. 1D, p=0.0003).

## Data Availability

Description: Quick Oil Red O software analysis package. Resource Type: Software. DOI: https://doi.org/10.22002/xwjnh-w6k67
